# Chronicle of a Soil Bacterium: *Paenibacillus polymyxa* E681 as a Tiny Guardian of Plant and Human Health

**DOI:** 10.3389/fmicb.2019.00467

**Published:** 2019-03-15

**Authors:** Haeyoung Jeong, Soo-Keun Choi, Choong-Min Ryu, Seung-Hwan Park

**Affiliations:** ^1^Infectious Disease Research Center, KRIBB, Daejeon, South Korea; ^2^Department of Biosystems and Bioengineering, KRIBB School of Biotechnology, Korea University of Science and Technology, Daejeon, South Korea

**Keywords:** PGPR, antimicrobial peptides, induced systemic resistance, polymyxin, non-ribosomal peptide synthetases, polyketides

## Abstract

The Gram-positive rhizosphere bacterium *Paenibacillus polymyxa* promotes plant growth and produces various antibiotics. Herein, we review research on this species over the past two and a half decades, and focus on the mechanisms of *P. polymyxa* strain E681, isolated from barley roots in the South Korea in 1995. Strain E681 has outstanding growth-promoting effects on barley, cucumber, pepper, sesame, and *Arabidopsis thaliana* and produces antimicrobial compounds that protect plants against pathogenic fungi, oomycetes, and bacteria. Induced systemic resistance elicited by treating seeds or roots with strain E681 is a possible mechanism for protecting systemic plant tissues from biotic and other environmental stresses. Genome sequencing has broadened our horizons for antibiotic development and other industrial applications beyond agricultural use. At least six gene clusters for the biosynthesis of antibiotics have been discovered, including polymyxin (*pmx*), which was recently re-instated as an antibiotic of last resort against Gram-negative drug-resistant bacteria. Three groups of antibiotic synthetases include the gene clusters that encode one for the non-ribosomal peptide polymyxin, fusaricidin, and tridecaptin, another for the lantibiotic paenilan, and the third for a polyketide. We successfully introduced the *pmx* gene cluster into the surrogate host *Bacillus subtilis* and created polymyxin derivatives by domain swapping. Furthermore, various E681 derivatives, including a high fusaricidin producer and strains lacking multi-antibiotics production, have been constructed by random mutagenesis and genome engineering. Thus, E681 is an important bacterium that contributes to both plant and human health.

## Introduction

A recent paradigm shift upon revisiting the role of soil microbes in plant health suggests that the rhizosphere microbiome is a key determinant of plant health (Berendsen et al., 2012). Plant growth-promoting rhizobacteria (PGPR), which colonize plant roots, draw special attention due to their beneficial effects on crops and ecosystems (Kloepper et al., 2004). Rhizosphere bacteria strains of various genera are designated PGPR. The fluorescent pseudomonads have received special interest due to their versatile metabolism, rapid growth, and strong mobility (Bakker et al., 2007). *Streptomycetaceae* are exceptional antibiotic producers and can suppress plant pathogens. However, the major barriers to agricultural usage of *Streptomycetes* spp. and *Pseudomonas* spp. are difficulties associated with mass production in liquid culture, and poor long-term storage due to a short shelf-life, respectively (Emmert and Handelsman, 1999). *Bacillus* spp. and *Paenibacillus* spp. are strong candidates for overcoming the drawbacks of *Streptomycetes* spp. and *Pseudomonas* spp. (Palaniyandi et al., 2013). *Bacillales* produce diverse antimicrobial peptides that suppress the growth and fitness of human and plant pathogenic microbes (Ongena and Jacques, 2008; Sumi et al., 2015). Additionally, other metabolites from *Bacillales* species include human immune activating factors and plant growth regulators (Farag et al., 2013; Levy et al., 2016; Postler and Ghosh, 2017; Sharifi and Ryu, 2018). Among *Bacillales* species, *Paenibacillus* spp. have received relatively less attention, since most research has focused on *Bacillus* species. Due to emerging issues with the re-use and side effects of last resort polymyxin antibiotics, and the occurrence of insects resistant to *Bacillus thuringiensis* toxins, we recently turned our attention to other *Bacillales* species.

Species belonging to the *Paenibacillus* genus were previously re-classified under the genus *Bacillus*, based on morphological characteristics. However, 16S rRNA sequence analysis (Ash et al., 1991) and PCR probe tests suggested that a group of eleven species should be considered a new genus, *Paenibacillus*, of which *P. polymyxa* is the type strain (Ash et al., 1993). In addition, *P. polymyxa* (formerly known as *Bacillus polymyxa*), is famous for its production of antimicrobial lipopeptides polymyxins, which were described as early as the 1940s and demonstrated to have very strong growth inhibitory activity against Gram-negative bacteria (Storm et al., 1977). In nature, the habitat of *Paenibacillus* spp. is mainly soil. Due to their multiple beneficial effects, *Paenibacillus* spp have implications for agriculture, environmental remediation, and even human and animal health. A recent review (Grady et al., 2016) presented a comprehensive summary on research history, scientific impacts, possible applications, and future prospects of *Paenibacillus*. The description of the genus *Paenibacillus* was recently amended following the isolation of *P. tumbae* sp. nov. (Huang et al., 2017). There are 230 approved species in the genus *Paenibacillus* according to the List of Prokaryotic Names with Standing in Nomenclature (LPSN^1^), and 342 genome sequences are available from the RefSeq database, including 133 genomes without species names as of 17 October 2018.

Out *Paenibacillus* spp., the type strain *P. polymyxa*, is regarded as a potential PGPR with a broad host range (Timmusk et al., 2005). One major mechanism of the plant health protection activity of certain *P. polymyxa* strains is the control of plant pathogenic fungi, oomycetes and bacteria by the production of antibiotic compounds (Raza et al., 2008) such as non-ribosomally synthesized lipopeptides polymyxins and fusaricidins. Beyond plant protection, lipopeptides from *Paenibacillus* spp. have great potential for the treatment of multidrug-resistant (MDR) and human pathogenic bacterial infections (Cochrane and Vederas, 2016). The expansion of publicly available bacterial genome sequences and the development of various genome mining methods (Ziemert et al., 2016) have enabled a systematic approach for the identification of novel and uncharacterized lipopeptides and polyketides from genome sequences (Aleti et al., 2015) and expanded the classical comparative genomic analyses of early available genome sequences, which were restricted to only four *P. polymyxa* strains E681, CR1, M1, and SC2 (Eastman et al., 2014).

This review presents and discusses current knowledge of the *P. polymyxa* strain E681, a representative strain belonging to the *P. polymyxa* species, including its strain isolation, plant growth-promoting activities, protectant effects, antibiotic production, and genome analysis. The characteristics of strain E681 are compared with those of other *P. polymyxa* strains. In particular, we focused on the identification of antimicrobial compounds and the development of high-yielding strains derived from genome engineering during the 22 years since strain E681 was first isolated in 1995. To the best of our knowledge, E681 is the most studied microorganism belonging to the genus *Paenibacillus*, and has been consistently studied by KRIBB^2^ researchers and collaborators in the Republic of Korea (South Korea). Furthermore, the most characterized strain for biosynthetic gene clusters (BGCs) for secondary metabolites as well as their final products among sequenced *Paenibacillus* strains. Here, we discuss strain E681 in relation to its interactions with plants, and its genome sequencing and comparative genomics, antimicrobial compound production, and industrial applications. The genome sequences of three new *P. polymyxa* isolates and two type strains of *Paenibacillus* spp., determined by South Korea group, could help expand the phylogenomic perspective of *P. polymyxa* and related species, including BGC diversity. Average nucleotide identity (ANI)-based analysis suggests that strain E681 should be classified as a novel species along with other strains that have *P. polymyxa* or other species names.

## Isolation and Characterization of *Paenibacillus polymyxa* Strain E681

**FIGURE 1:**
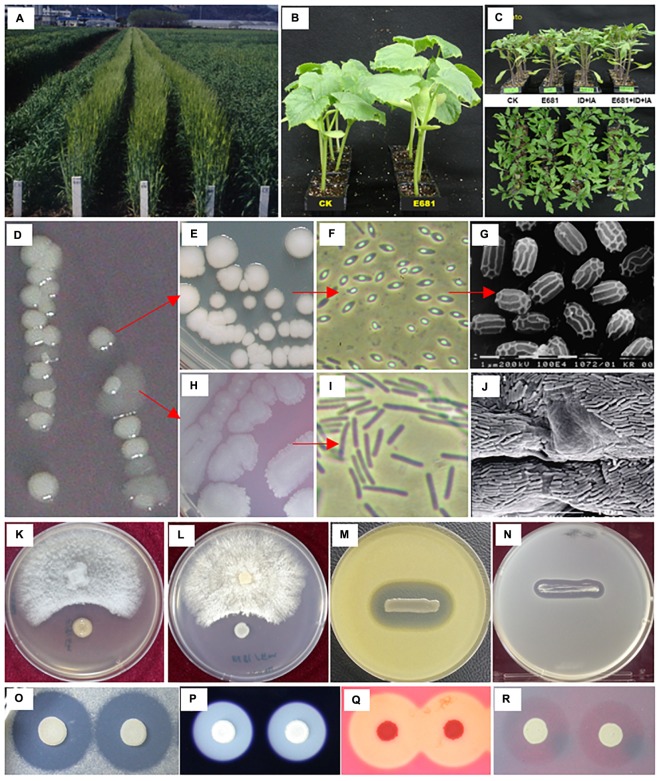
Characteristics of *Paenibacillus polymyxa* E681 and its plant-beneficial traits. Growth promotion of barley (Cho et al., 1999), cucumber, and tomato by treatment with E681 spores (**A–C**, respectively); natural phenotypic variation of E681 colonies during growth on tryptic soy broth agar (TSA) plates **(D)**; typical colonies of E681 **(E)**; light and electron microscopy observations of E681 spores (**F,G**, respectively); flat variant colonies of E681 **(H)** and light microscopy observation of cells of variant colonies **(I)**; scanning electron microscopy of E681 colonies in cucumber roots **(J)** (Choi et al., 2004); antimicrobial activity of E681 against *Fusarium oxysporum* **(K)**, *Rhizoctonia solani* **(L)**, *Micrococcus luteus* **(M)**, and *Escherichia coli* **(N)**; extracellular enzyme activities of protease **(O)**, amylase **(P)**, cellulose **(Q)**, and mannanase **(R)**. Seeds were soaked with 10^7-9^ E681 spores and cells used to assess the plant growth-promoting and root-colonizing abilities of E681. To test antifungal activity, mycelial plugs of fungal pathogens and pre-grown E681 cells were spot-inoculated on a potato dextrose agar plate. To test antibacterial activity, pre-grown E681 cells were streaked on TSA pre-inoculated with bacterial cells at a final concentration of 10^6^ cfu/ml. The extracellular enzyme activities were analyzed by spot-inoculating E681 cells onto TSA plates containing substrates.

As mentioned above, *P. polymyxa* strains have been mostly isolated from soil and rhizosphere, the presumed natural habitats of *P. polymyxa*. For instance, the model strain *P. polymyxa* E681 was isolated from roots of winter barley in South Korea (Ryu and Park, 1997; Ryu et al., 2005b). Among 3179 endospore-forming bacteria isolated after heat treatment at 80°C for 1 h, 31 isolates antagonistic against oomycetes pathogens, particularly against *Pythium* spp. and *Phytophthora* spp. were obtained. Strain E681 was eventually selected based on its aggressive root colonization capacity compared with other *Bacillales* species (Choi et al., 2004), and its strong antagonism against a broad spectrum of fungi, oomycetes, and bacteria *in vitro*. As shown in Figure 1, our previous studies demonstrate that strain E681 promotes plant growth when seeds are soaked in bacterial suspension, displays antagonism against plant pathogenic fungi, oomycetes, and bacteria, produces various extracellular hydrolytic enzymes, and exhibits potent root colonization activity of various crop species under soil conditions (Ryu and Park, 1997; Choi et al., 2004; Cheong et al., 2005; Ryu et al., 2005a,b). The production of extracellular hydrolytic enzymes by strain E681 indicates that it has potential for use in industrial applications and as a biological control agent against plant pathogenic fungi and bacteria. Furthermore, strain E681 secretes diverse hydrolytic enzymes including proteases, amylases, cellulases, and mannanases, which assist bacterial competence in rhizosphere (Bach et al., 2016; Figures 1O–R). Additionally, such enzymes are recognized to have important industrial applications (Grady et al., 2016). Because it has great potential in agriculture, biomedicine, and industry, E681 was the first strain to have its genome completely sequenced, under the original label of *P. polymyxa* (Kim et al., 2010).

It is also worth noting that *P. polymyxa* strains often display a diverse colony shape when grown on complex agar media. Typically, colony formation on solid medium results in a variable morphology, referred to as phase variation or phenotype variation without genetic alteration (Figure 1D,E,H). Once the colony shape changes to a flat form, spores cannot be formed under normal culture conditions (Figure 1I) and antimicrobial activity and extracellular enzymatic activity are significantly reduced (data not shown). Initially, colonies with different shapes were suspected to be contaminants, but genome sequencing detected no differences in nucleotide sequence between the genomes of the two colony morphology types, indicating that they were identical (data not shown).

Additionally, the production of volatile compounds is a key factor mediating inter-kingdom interactions, leading to the elicitation of induced systemic resistance (ISR) in plants (see section “Interactions With Plants and Soil Microorganisms”). To understand the genome functions responsible for adaptation to a plant-associated lifestyle, and to prospect for potential metabolites and gene products that can be used for agricultural and biomedical purposes, the complete genome sequence was determined using conventional Sanger sequencing methodology (see section “Genomics of *P. polymyxa* E681”). Genome sequence information revealed that E681 harbors an array of BGCs for both known and unknown compounds. At least five of these have since been characterized in terms of both the chemical structures of the compounds and BGC organization (see section “Antimicrobial Compound Production”).

## Interactions With Plants and Soil Microorganisms

In this section, we summarize evidence showing that *P. polymyxa* E681 promotes plant growth and protects against plant pathogens, and compare its properties with those of other strains of *P. polymyxa*. The possible mechanisms underpinning plant responses to *P. polymyxa* are also discussed.

### Promotion of Plant Growth

In early studies on *P. polymyxa* in the mid-1990s, many scientists explored whether *P. polymyxa* promotes plant growth. Seed treatment with *P. polymyxa* on cucumber as a model crop increased shoot fresh weight by 60% compared with water controls in greenhouse experiments (Ryu and Park, 1997; Ryu et al., 2005b). Furthermore, drenching soil with 10^8-9^ colony-forming units (CFU)/ml, strain E681 increased height, fresh weight, and dry weight by 3-38% in 3-, 4-, and 5-week-old pepper seedlings under greenhouse and field conditions (Hahm et al., 2012). Field trials on sesame plants were also conducted by soaking seeds with *P. polymyxa* strain E681, which improved the yield by 2.85-fold vs. water controls (Ryu et al., 2006). Similarly, seed treatment with strain E681 increased seedling growth and grain yield in barley and wheat after overwintering (Ryu and Park, 1997). Besides crop plants, strain E681 enhanced total leaf surface area in the model plant *Arabidopsis thaliana in vitro* [on Murashige and Skoog (MS) agar medium] (Ryu et al., 2005a). In the same study, in two separate experiments (direct seeding with vernalized seeds, and 4-week-old seedlings), treating soil with E681 improved foliar fresh weight by more than 30% vs. water controls (Ryu et al., 2005a). Similarly, plant growth promotion was observed when *A. thaliana* seeds were directly seeded in soil amended with 10^8^ CFU/ml E681; fresh weight reached 218 mg at 3 weeks after seeding, compared with only 50 mg for controls (Ryu et al., 2005a). Later, soil drenching application of strain E681 achieved similar effects on plant growth (Kwon et al., 2016).

The mode of action of PGPR-mediated plant growth promotion, including that mediated by *P. polymyxa*, has been investigated. Two mechanisms have been advanced: (1) Direct plant growth promotion via bacterial secretion of mimic phytohormones and bacterial nitrogen fixation and (2) Indirect plant growth promotion via PGPR suppression of plant pathogens that cause plant disease. Previous studies demonstrated that siderophore production plays an important role in plant growth promotion and biocontrol. However, genome-wide analysis of strain E681 failed to reveal siderophore non-ribosomal peptide synthetase (NRPS) genes (data not shown).

#### Mimic Phytohormone Secretion

Previously mimic plant hormones secreted from PGPR were reported (Lugtenberg and Kamilova, 2009). Plant hormones released by *P. polymyxa* strain E681 include auxins such as indole-3-acetic acid (IAA). Auxins and their intermediates including IAA synthesized by PGPR are key determinants of plant growth stimulation. However, in *Paenibacillus* spp., little is known about the genetic basis of the key enzymes responsible for IAA biosynthesis. Ethyl acetate extraction and high-performance liquid chromatography (HPLC) analysis have revealed that the IAA and tryptophol (TOL) contents of E681 are 11.84 and 2.30 μg/ml, respectively (Phi et al., 2008b). Analysis of IAA intermediates revealed the presence of indole-3-pyruvic acid (IPA), indole-3-aldehyde (IAAld), indole-3-lactic acid (ILA), and TOL. Comparative genome analysis of strain E681 with other *P. polymyxa* strains revealed four genes that synthesize IPA, an IAA intermediate. IPA production was demonstrated in *Escherichia coli* BL21(DE3) heterologously expressing the IPA decarboxylase (*ipdC*) gene from the E681 genome (Phi et al., 2008a). However, even though strain E681 was shown to secrete IAA into the culture media, whether plants perceive IAA or its intermediates, and whether this resulted in activation of plant hormonal signaling pathways, remained unknown. Recent proteome analysis of *A. thaliana* seedlings treated with strain E681 revealed augmentation of tryptophan-derived IAA biosynthesis pathway-related proteins ASB1, GSTF6, CYP71B15, and NIT1, which catalyze production of indole-3-acetaldoxime (IAOx) (Kwon et al., 2016). Furthermore, liquid chromatography-mass spectrometry (LC-MS) confirmed indole-3-acetonitrile (IAN) and IAA production following activation of protein expression in strain E681. Genetic evidence for the modulation of endogenous IAA signaling was also confirmed by the auxin-insensitive *Arabidopsis eir1-3* mutant (Ryu et al., 2005a). Taken together, this evidence indicates that auxins released by E681 play an important role in E681-elicited plant growth promotion. Additionally, cytokinins, pyrroloquinoline quinone (PQQ), and long-chain bacterial volatiles are candidate bacterial factors for plant growth promotion by strain E681 (Seul et al., 2007; Lee et al., 2012). Interestingly, bacterial proteome analysis following barley root exudate stimulation of ABC transporters indicated activation of protein secretion in a plant signal-dependent manner (Seul et al., 2007). However, the exact amounts of plant growth regulators, such as plant hormones, present in the rhizosphere following colonization of the plant root system by E681 are yet to be measured *in situ* directly, and whether the quantities of metabolites in root exudates are sufficient for initiating auxin signaling in plants remains unknown.

#### Non-symbiotic Nitrogen Fixation

Free-living nitrogen fixation is a well-known mode of action on the growth promotion of rhizosphere *Bacillus* spp. and *Paenibacillus* spp. (Ruschel et al., 1975). Previously, nitrogen fixation by root-colonizing *P. polymyxa* was reported to be a major mechanism for the promotion of the growth of diverse plant species including lodgepole pine, wheat, sugarcane, and canola (Ruschel et al., 1975; Heulin et al., 1994; Timmusk et al., 2005; Bal and Chanway, 2012a,b; Padda et al., 2017). Despite recent advances in our understanding of symbiotic nitrogen fixation, such as rhizobia and mycorrhizae-legume plant symbiosis and non-symbiotic nitrogen fixation by free-living Gram-negative bacteria, *Azotobacter* spp., and *Azospirillum* spp., spore-forming Gram-positive bacteria-mediated nitrogen fixation has not been extensively investigated (Steenhoudt and Vanderleyden, 2000; Schmitz et al., 2002; Mus et al., 2018). The gene cluster that confers nitrogen fixation in *P. polymyxa* could provide a good model for understanding the mechanisms of nitrogen fixation by free-living spore-forming Gram-positive bacteria.

#### Emission of Bacterial Volatile Compounds

It is also noteworthy that metabolic cross-talking occurs between exopolysaccharide (EPS) and 2,3-butanediol biosynthesis (Okonkwo et al., 2018). Previously, bacterial volatile compounds emitted from strain E681 into the headspace of flasks were shown to enhance seedling growth of *A. thaliana* (Seul et al., 2007; Lee et al., 2012). The major bacterial volatile compounds from strain E681 were 2,3-butanediol and tridecane, a C13 long-chain fatty acid volatile. *P. polymyxa* strain DSM 365, a known efficient producer of 2(R),3(R)-butanediol, has its genome completely sequenced (Xie et al., 2015). From the industrial point of view, it is noteworthy that *P. polymyxa*, a non-pathogenic 2,3-butanediol producer, is one of the rare organisms that produces the R form isomer of 2,3-butanediol in pure form without the need for genetic engineering. By contrast, *Klebsiella pneumonia*, a serious human pathogen, considered a promising organism for the mass production of 2,3-butanediol (Garg and Jain, 1995). However, the pathogenic characteristics of encapsulated *K. pneumonia* are considered an obstacle to its industrial applications (Jung et al., 2013). 2,3-Butanediol is an important intermediate metabolite that modulates the secretion of bacterial secondary compounds by stabilizing biosynthesis-related enzymes (Garg and Jain, 1995). Recently, metabolic engineering of this strain to disrupt the *sacB* gene encoding levansucrase, the enzyme responsible for the biosynthesis of the major EPS of *P. polymyxa*, levan, resulted in up to a 27% increase in 2,3-butanediol production when it was grown on sucrose (Okonkwo et al., 2018). These data indicate that increased bacterial volatile production can be used to improve PGPR capacity on plant growth promotion. Similar to other bacterial species, *P. polymyxa* EPS plays a critical role in the root colonization of peanut plants and elicits plant defense against crown rot disease (Haggag, 2007). Discovering the antagonistic regulatory mechanisms of EPS production and bacterial volatile 2,3-butanediol production led to modulating *P. polymyxa*-mediated plant growth and health improvements. Rütering et al. (2017) characterized the complete gene cluster (∼34 kb) for the biosynthesis of heteropolysaccharides, another kind of EPS, from DSM 365 (Rütering et al., 2018), which appears to be widely distributed among *Paenibacillus* species (see section “Requirement for a New Phylogenomic Position for Strain E681 and Other Related *Paenibacillus* spp. Strains”). Fine-tuning of bacterial physiology though the engineering of bacterial regulatory networks will help maximize the contribution of *P. polymyxa* to crop productivity.

### Suppression of Plant Pathogens

Spore-forming bacteria isolated from winter barley and wheat were shown to inhibit diverse plant pathogenic fungi, oomycetes, and bacteria, and exhibited promising biological control potential under field conditions (Ryu et al., 2005b). Following isolation of *P. polymyxa* E681, strong suppression of soil-borne pathogens *Pythium ultimum*, *Rhizoctonia solani*, and *Fusarium oxysporum* was detected *in vitro*, accompanied by a significant reduction in damping-off symptoms caused by these oomycetes and fungal pathogens (Ryu et al., 2005b). In this study, pelleting sesame seeds with strain E681 and pelleting materials such as clay and talc improved seed germination under greenhouse and field conditions. Field trials demonstrated increased health by 92%, compared with 24% for controls that suffered heavy fungal infection, leading to reduced health and crop yield (Ryu et al., 2006). At the end of the growing season, the sesame yield of plants pelleted with strain E681 was 77 kg/1000 m^2^, compared with only 24 kg/1000 m^2^ for the conventional treatment. Thus, strain E681 is a potent biological control agent under both greenhouse and field conditions, and the biocontrol mechanism can be considered to be caused by the production of a variety of antimicrobials discussed in detail below (see section “Antimicrobial Compound Production”) and extracellular hydrolytic enzymes production (see section “Isolation and Characterization of *Paenibacillus polymyxa* Strain E681”; Figure 1).

### Augmentation of the Plant Immune System

Systemic activation of plant innate immunity elicited by PGPR. ISR capacity against diverse pathogens of numerous crop and model plant species has been reported for many PGPR. The minimum criterion for ISR is spatial separation between inoculation of PGPR and the site of pathogen challenge (Kloepper et al., 2004). In 2012, soil drenching with a bacterial suspension of strain E681 protected pepper plants against bacterial spot disease caused by *Xanthomonas axonopodis* pv. vesicatoria on aboveground parts (foliar parts such as stems and leaves) (Hahm et al., 2012). Due to limited genetic and molecular knowledge on pepper plants, despite the option of gene knock-down techniques such as virus-induced gene silencing, *A. thaliana* was employed as a model plant to perform genome-wide whole-proteome analysis using two-dimensional electrophoresis (2DE) in conjunction with matrix-assisted laser desorption-ionization-time of flight (MALDI-TOF) MS (Kwon et al., 2016). Overall, root colonization by strain E681 yielded 17 and 24 differentially expressed protein spots in roots and shoots, respectively. Subsequent transcriptome and metabolite production analyses confirmed that strain E681 modulates the expression of PDF1.2 and camalexin proteins involved in jasmonic acid (JA) signaling, and contributes to ISR capacity against necrotrophic pathogens such as *Botrytis cinerea* (Kwon et al., 2016). Additional abiotic stress-related proteins and antioxidant enzymes including GST, GPX8, APX1, PER43, and CML42 were also induced by treatment with strain E681, indicating enhanced systemic tolerance against environmental stresses including drought, salt, and cold (Yang et al., 2009).

#### Involvement of Auxin in ISR

Similar to the mechanism underpinning plant growth promotion, strain E681 elicits ISR via volatile compounds and auxins (Seul et al., 2007; Phi et al., 2008b; Lee et al., 2012; Park et al., 2013). As described above, large amounts of IAA and its intermediate IPA are released from E681 (Seul et al., 2007; Phi et al., 2008b). The IAA-related pathway contributes not only to plant growth, but also to plant defenses via. camalexin biosynthesis (Fu and Wang, 2011). In the *A. thaliana* root system, strain E681 induces ASB1, GSTG6, and CYP71B15, which are related to the tryptophan-derived camalexin biosynthesis pathway (Kwon et al., 2016).

#### Production of Bacterial Volatiles, Tridecane and 2,3-Butanediol

Strain E681 also produces volatile compounds that elicit ISR against *Pseudomonas syringae* pv. tomato and *P. syringae* pv. maculicola in *A. thaliana* (Lee et al., 2012). Of 30 volatile compounds, 10 mM and 100 μM C13 tridecane was found to be largely responsible for priming plant immunity, and pretreatment with 10 mM tridecane and strain E681 volatiles induced *PR1* and *VSP2* expression earlier and more strongly following pathogen challenge. These phenomena are referred to as “defense priming” and “plant memory” (Crisp et al., 2016). We also demonstrated that E681-derived long hydrocarbon hexadecane triggered an ISR response that was stronger than the response triggered by acetoin or 2,3-butanediol (Park et al., 2013). Furthermore, pre-application of E681 on pepper roots increases plant insect susceptibility against aphid leaf infestation (Kim et al., 2016). More recently, *P. polymyxa* strain DSM 365 was shown to increase strongly ISR against an oomycetes pathogen *Phytophthora parasitica* (Park et al., 2018). The major bacterial determinant for inducing ISR was 2,3-butanediol. Direct leaf infiltration with 2,3-butanediol protected tobacco plants against *Phytophthora parasitica* in the root system through down-regulation of reactive oxygen species biosynthesis-related genes and pathogenesis-related (*PR*) gene expression (Park et al., 2018). As described above (Okonkwo et al., 2018), engineered DSM 365, an 2,3-butanediol producer, can be used to improve ISR against diverse pathogens. Thus, collectively, evidence suggests that strain E681 strongly modulates the plant immune system. Analysis of E681-elicited ISR should be expanded to other crop plants in greenhouse and field trials in the future.

### Interactions With Fungi

In soil and rhizosphere environments, interactions between PGPR and other soil microorganisms are complex and can affect the beneficial effects of the bacterium. In a study on the interactions between *P. polymyxa* E681 and fungi, it was found that *Penicillium citrinum* could induce swarming motility in *P. polymyxa* (Park et al., 2008). We found that citrinin, a mycotoxin produced by the fungus, was involved in the induction of *P. polymyxa* motility via transcriptional activation of genes related to the expression of flagella. This finding provides insight into the interaction mechanisms among microorganisms in natural environments.

## Genomics of *P. polymyxa* E681

### Genome Sequencing of *P. polymyxa* E681

In the 1990s, our group participated as a member of the international consortium in genome sequencing of the Gram-positive model organism *Bacillus subtilis* (Kunst et al., 1997) using classical clone-based Sanger sequencing methodologies. Despite a positive outcome and compatibility with genomic research on the genus *Bacillus* and related microbes, obtaining complete bacterial genomes remained challenging until the advent of next-generation sequencing technologies that became available in the early 2000s. Complete genome sequencing of *P. polymyxa* E681 (Kim et al., 2010) was one of the earlier government-funded genome research programs (Jeong et al., 2008), performed in collaboration with GenoTech Corporation (Daejeon, South Korea). Because the first version of the complete genome sequence, which lacked plasmid sequences, obtained in April 2005, was based on only 6.7-fold coverage (∼62,000 chromatograms) produced using plasmid/fosmid/BAC libraries, the error rate was as high as 10.74 errors/10 kb. Sequencing errors located in low-quality regions were gradually corrected using manual PCR/Sanger sequencing, and finally, in 2009, Illumina GA IIx sequencing (76 bp single-ended sequences, totaling 2.4 Gb) was employed to dramatically improve sequence quality. Read mapping and variant detection were conducted using the MAQ package (Li et al., 2008), followed by confirmatory PCR/Sanger sequencing, and the resulting genome sequence was the first to be publically released for strain E681 (GenBank CP000154.1, 5,394,884 bp, September 2010). The sequence was updated in July 2013 (CP000154.2), in which a T(17) → T(16) correction rectified a false frameshift at *spoIIIAF* (PPE_02850, now renumbered as PPE_05995). When Illumina reads were re-mapped onto CP000154.2 using the current version of CLC Genomics Workbench (11.0), no variants were found, implying high confidence for the current release of the E681 genome.

Before the public release, preliminary in-house genome annotation was circulated internally to help identify genes responsible for plant interactions or antibiotic production. For the second version (CP000154.2), the NCBI Prokaryotic Genome Annotation Pipeline (PGAP) was employed and genes were edited based on comparison with the previous version. A minor annotation update without genome sequence correction was made in January 2018 to assign gene and product names systematically to those involved in the biosynthesis of antimicrobial secondary metabolites.

### Requirement for a New Phylogenomic Position for Strain E681 and Other Related *Paenibacillus* spp. Strains

Early taxonomic allocation of E681 to *P. polymyxa* species was solely based on 16S rRNA gene sequence analysis. However, recent species demarcation approaches allow strains sharing genome similarity with the type strain of a particular species above a certain criterion to be assigned that species name. Genome similarity is often expressed as ANI or other measures (Goris et al., 2007; Richter and Rossello-Mora, 2009), and 95 or 96% pairwise ANI values are commonly adopted as threshold values. Since no other genomes for *P. polymyxa* species were available when working on the draft genome sequence of strain E681, our group determined genome sequences for two other type strains, namely, *P. polymyxa* ATCC 842^T^ (Jeong et al., 2006, 2011) and *P. peoriae* KCTC 3763^T^ (Jeong et al., 2012), for comparison with that of E681. The latter was chosen because the 16S rRNA sequence of E681 is most similar to that of the *P. peoriae* type strain (99.46%). Unexpectedly, the ANI score between E681 and ATCC 842^T^ calculated using JSpecies (Richter and Rossello-Mora, 2009) was only 86.66%, which implied that E681 may belong to another *Paenibacillus* species. By the end of 2012, two more complete genome sequences of *P. polymyxa* species became available, namely, those of SC2 (Ma et al., 2011) and M1 (Niu et al., 2011). While these two strains were very similar in terms of ANI score (>99.8%) and overall genome organization, they were distinct from both E681 and ATCC 842^T^. Specifically, the ANI value between E681 and M1 (and also SC2) was below 90%. In other words, no genomes with the original label of *P. polymyxa*, available before 2012, could be assigned to the same species as E681. Thus, we had to await the availability of additional genomes to accurately determine the phylogenetic position of E681. Since then, the genomes of *P. polymyxa* var. colistinus KCCM 40454 (=ATCC 21830; POVT01), *P. polymyxa* F4 (POVS01), and *P. peoriae* HS311 (CP011512-3) have been sequenced by our group to expand current knowledge on *Paenibacillus* strains for plant probiotic usage, and for the discovery of novel antimicrobial compounds. The first two strains have been used for the identification of BGCs for polymyxin A and E (also known as colistin) (Park et al., 2012b), respectively, and strain HS311, which possesses potent antifungal activity, has been successfully used to increase potato yield (Park et al., 2015).

**FIGURE 2:**
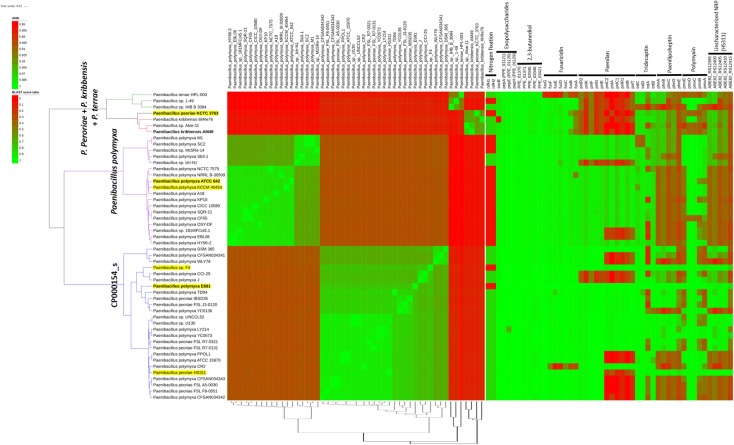
Comparative genomic analysis of 50 selected *Paenibacillus* strains. All available genome sequences, identified by TrueBac ID (https://help.ezbiocloud.net/user-guide/truebac-id/about-truebac-id/) and belonging to the species *P. polymyxa* (18 strains), “CP000154_s” (25), *P. peoriae* (1), *P. kribbensis* (3), and *P. terrae* (3), were downloaded from EzBioCloud and BLASTN-based pairwise ANI values were calculated using pyani (https://github.com/widdowquinn/pyani). Species were chosen whose 16S rRNA gene sequences were similar to those of E681 (>96% similarity). Type strains and E681 are shown in boldface; strains in a yellow background represent those sequenced by our group; and strains grouped in the same species in EzBioCloud have the same clade colors. The left heatmap shows the ANI matrix, where rows are sorted according to the complete linkage clustering data calculated in the R environment (hclust function) and columns are sorted as they appear in dRep (Olm et al., 2017) primary clustering (dendrogram shown at the bottom of the heatmap). The BLAST score ratio matrix shown in the right heatmap shows the presence of selected genes across strains using the LS-BSR method (Sahl et al., 2014). *nifH* sequences and genes starting with ABE82 were collected from *P. durus* ATCC 35681 (AJ515294.1 and AJ299453.1) and *P. peoriae* HS311 (CP011512.1), respectively. All other sequences were retrieved from strain E681. The figure was drawn using the iTOL server (Letunic and Bork, 2016).

It is well known that current prokaryotic species assignments in sequenced genomes in public databases may be inconsistent with correct species allocations. Recent efforts to reclassify prokaryotic species include EzBioCloud (Yoon et al., 2017), an integrated database of 16S rRNA genes and genome sequences. For example, EzBioCloud version 2018.10 declared CP000154_s as a candidate species (“phylotype”) for strains that were originally labeled as *P. polymyxa* (15 strains), *P. peoriae* (seven strains), and unidentified species (three strains). CP000154_s also incorporates strains E681, F4, HS311 (strains we previously isolated and sequenced) and DSM 365. Only 18 strains with the original *P. polymyxa* (including the type strain ATCC 842^T^) and *Paenibacillus* species labels were classified as “genuine” *P. polymyxa* species. ANI matrix visualization of all *P. polymyxa* and CP000154_s genomes available on EzBioCloud clearly indicates that they are clustered as two separate species groups (Figure 2). Subdivision of each group into several species might be possible depending on the ANI cutoff used, but this will require further phylogenetic analysis including investigation of core and accessory genes, as well as phenotypic characterization. This implies that strains under the original species name of *P. polymyxa* (38 genomes^3^) and other closely related ones might need to be subdivided into several novel species on the basis of contemporary analysis method for genomic relatedness.

Comparative analysis of *P. polymyxa* strains has revealed that nitrogenase reductase genes (*nifH*) could be used to discriminate CP000154_s from genuine *P. polymyxa* species but not from other strains such as E681 and F4, which *lack nifH* genes. Although *nifH* was detected using PCR in *P. polymyxa* type strain ATCC 842 (Achouak et al., 1999), the submitted *nifH* partial sequence (AJ223997) could not be found in the genome sequence of ATCC 842 (AFOX01) determined later by our group, suggesting the PCR result was an artifact. The presence and absence patterns of selected genes, responsible for nitrogen fixation, EPS production, 2,3-butanediol productions, and biosynthesis of antimicrobial compounds, are shown in the right heatmap in Figure 2. We found that genes involved in the production of EPS and 2,3-butanediol were widely distributed among the analyzed strains. It is also noteworthy that CP000154_s contains strains that have been extensively studied for plant interactions and biotechnological applications. This has also been the case for E681, DSM 365 (EPS and 2,3-butanediol production), and PPLO1 (=DSM 292). The latter strain was studied to see if it could be used as a heterologous expression system for secretory proteins because it has low exoprotease activity (Heinze et al., 2018).

## Antimicrobial Compound Production

**FIGURE 3:**
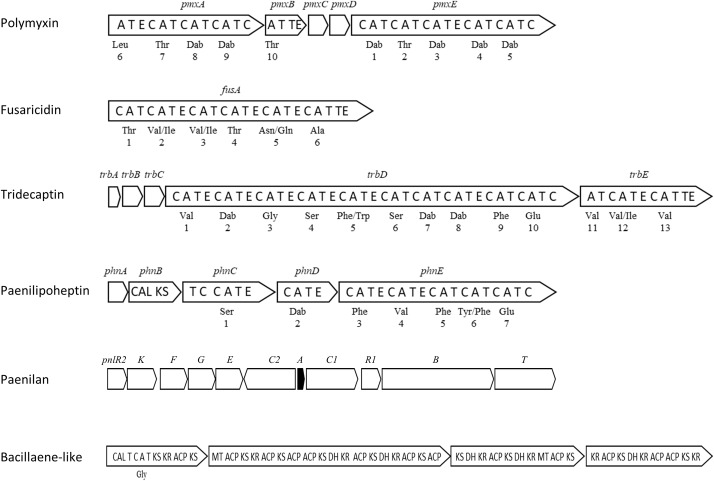
Antibiotic gene clusters in the genome of *P. polymyxa* E681 (not drawn to scale). Amino acids recognized by the corresponding A-domains are shown below the modules, and the numbers indicate the order of assembly of the amino acids. The *pnlA* gene (black) encodes a prepeptide of paenilan. Domains are coded as follows: A, adenylation; T, thiolation; E, epimerization; C, condensation; TE, termination; CAL, CoA ligase; KS, ketosynthase; KR, ketoreductase; ACP, acyl carrier protein; MT, methyl transferase; DH, dehydratase.

Genome sequencing of *P. polymyxa* strains revealed numerous antibiotic biosynthetic genes in the genome encoding NRPSs, polyketide synthases (PKSs), and bacteriocins. NRPS and PKS generally have a modular structure, with the modules containing multiple domains responsible for recognition, activation and condensation of an amino acid and an acyl-CoA (Aleti et al., 2015). An NRPS module typically includes at least one condensation (C) domain, an adenylation (A) domain, and a thiolation (T) domain. Other domains including those for epimerization (E) and termination (TE) domains also contribute to antibiotic synthesis. Figure 3 shows BGCs for antimicrobials characterized in strain E681. These diverse antibiotic production machineries presumably help to fight against competitors that are harmful to plants in the environment, making them useful for plant protection. In addition, some antibiotics have been used for medical purposes for the treatment of MDR bacteria. According to the antiSMASH database (Blin et al., 2017), the average number of BGCs predicted from 46 complete *Paenibacillus* genomes is 8.5 (11 for E681); however, this number differs depending on the strain (standard deviation 4.7; 1–17 per genome). NPRS was the most common BGC among 2747 BGCs found in the sequences of all sequenced *Paenibacillus* genomes, including both complete and incomplete sequences. Comparison of BGC numbers among species was not meaningful because half of the analyzed genomes were from strains from unidentified *Paenibacillus* species. Furthermore, current species allocation needs reconsideration as mentioned earlier.

### Polymyxins

#### Identification of Polymyxin Biosynthetic Gene Cluster

Polymyxins are cyclic heptapeptide antibiotics with fatty acid-acylated tripeptide side chains that possess excellent bactericidal activity against Gram-negative bacteria. There are various types of polymyxins depending on the fatty acid type and the composition of the 3rd, 6th, 7th, and 10th amino acids. Since their discovery in 1947, more than 30 types of polymyxins have been reported (Rabanal and Cajal, 2017), including polymyxin B and E (colistin) that have been commonly used in clinical practice since the late 1970s for the treatment of infections caused by Gram-negative pathogenic bacteria. However, serious side effects such as severe nephrotoxicity and neurotoxicity have restricted their application to use as an ointment on local surface wounds (Hermsen et al., 2003). However, recently, limited therapeutic options for treating MDR Gram-negative pathogens have led researchers to revisit these old antibiotics as agents of last resort for fighting MDR pathogens (Chung et al., 2016).

**FIGURE 4:**
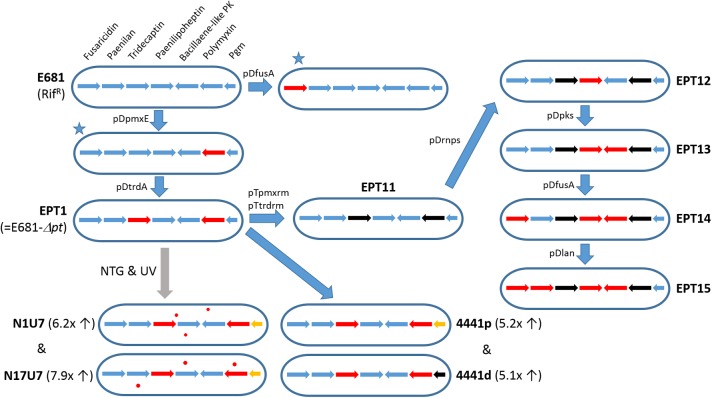
Development of E681 derivatives for the characterization of antibiotic biosynthesis genes. Arrows indicate *fusA*, *pnlA*, *trdA*, *phnE*, PPE_03011, *pmxA*, and PPE_04441 (*pgm*) genes. Red and black arrows represent genes disrupted using knockout plasmids and genes with null mutations, respectively. The initial two *fusA* or *pmxA* knockout mutants (marked with blue stars) were constructed for functional identification of antibiotic-producing gene(s) (Choi et al., 2008, 2009). The EPT1 strain was randomly mutagenized using *N*-methyl-*N’*-nitro-*N*-nitrosoguanidine (NTG) treatment and UV irradiation, yielding two separate mutants N1U7 and N17U7 (Kim et al., 2014). The fold increase in fusaricidin activity is shown in parentheses. Orange arrows denote Gln170Pro mutations in the *pgm* gene (Kim et al., 2014). Red dots represent mutations differentially occurring in each mutant strain. By introducing mutations only in the *pgm* gene in EPT1, fusaricidin production was enhanced (mutants 4441p and 4441d). Antibiotic biosynthetic genes in EPT11 were serially knocked out, leading to paenilan producer EPT14 (Park et al., 2017). Finally, EPT15 was constructed with no intact antibiotic genes and possessing no antimicrobial activity (Park et al., 2017).

The first NRPS gene cluster for polymyxin (*pmx*) synthesis was identified in *P. polymyxa* E681 by Choi et al. (2009). This gene cluster includes five ORFs, designated *pmxA*, *pmxB*, *pmxC*, *pmxD*, and *pmxE*. Analysis of PmxA (4953 amino acids), PmxB (1102 amino acids), and PmxE (6312 amino acids) revealed four, one, and five modules, respectively, and the prediction that together they synthesize polymyxin A (Ansari et al., 2004). Polymyxin A synthesis was indeed confirmed by LC-MS analysis of wild-type E681 and *pmxE* knockout mutant EPT1 (Figure 4). PmxC (608 amino acids) and PmxD (577 amino acids) were proposed to be membrane transporters, and their amino acid sequences share 40.5 and 43.5% identity, respectively, with TycD and TycE members of the ABC transporter family of *Brevibacillus brevis* (Mootz and Marahiel, 1997). After the discovery of the polymyxin A gene cluster, several other polymyxin biosynthetic genes involved in the synthesis of polymyxins B, E, and P have been identified (Shaheen et al., 2011; Park et al., 2012b; Niu et al., 2013; Tambadou et al., 2015). Sequence analyses showed that polymyxin biosynthetic genes share >90% amino acid sequence homology, and their genetic organization is highly conserved, even in other *Paenibacillus* species.

#### Heterologous Expression of Polymyxin

Restrictions on polymyxin use due to its deleterious effects on human organs have encouraged the development of low-toxicity polymyxin derivatives. Although chemical and enzymatic modifications have been reported (Rabanal and Cajal, 2017), production of diverse derivatives has been limited by the structural complexity of polymyxins, and genetic approaches may be more successful since the gene sequences are accessible. In general, the development of new antibiotic derivatives through genetic engineering in antibiotic-producing wild-type strains is difficult due to their low transformation efficiency. For example, heterologous expression of the *pmx* gene cluster of *P. polymyxa* E681 has been achieved in *B. subtilis* 168, a well-known strain that is easy to genetically manipulate (Choi et al., 2009). The gene cluster was integrated into the *amyE* locus of *B. subtilis* 168 in which *sfp* function was restored, resulting in polymyxin production only in the presence of extracellularly added L-2,4-diaminobutyric acid (L-Dab), because *B. subtilis* does not possess a Dab synthesis mechanism and polymyxin is a Dab-rich antibiotic. Dab synthesis is mediated by 2,4-diaminobutyrate aminotransferase encoded by *ectB*. Generally, *ectB* is part of the *ectABC* operon responsible for ectoine synthesis (Saum and Muller, 2008). However, no homologs of *ectA* or *ectC* have been found in the genome of *P. polymyxa*. The genetic background suggests that Dab may not be used as an intermediate for ectoine synthesis, resulting in accumulation of Dab in the cell, which would be advantageous for polymyxin synthesis. A *B. subtilis* strain that could produce polymyxin without exogenous Dab was subsequently generated by introducing the *ectB* gene from *P. polymyxa* E681 (Park et al., 2012c). Additionally, expression of the *pmx* gene cluster in *B. subtilis* was completely inhibited in the *spo0A* mutant. However, in the *abrB* knockout and *abrB-spo0A* double knockout organisms, polymyxin production was increased, indicating that AbrB represses *pmx* gene expression. Furthermore, AbrB was confirmed to directly bind to the *pmxA* promoter region using electrophoretic mobility shift assays.

#### Synthesis of New Polymyxin Derivatives by Domain Swapping

Besides chemical modification, early efforts to develop non-ribosomal peptide antibiotic derivatives relied on obtaining an exogenous supply of modified amino acid precursors, endogenous engineering of precursor biosynthesis, and enzymatic tailoring such as halogenation, glycosylation, acylation, and sulfation. Since NRPSs have a modular structure, module and domain swapping methods have been used to generate novel peptide derivatives (Schneider et al., 1998; Miao et al., 2006). Recently, genetic engineering technologies such as directed evolution, synthetic biology, and CRISPR-Cas9-based gene editing have provided promising tools for the development of novel peptide antibiotics (Winn et al., 2016). However, many wild-type strains producing antibiotics are difficult to genetically manipulate, and many antibiotic genes are silent in native strains, representing bottlenecks for these genetic approaches. In a previous study, a conceptual experiment was carried out to generate polymyxin derivatives by domain swapping in the surrogate host *B. subtilis* (Kim et al., 2015). Polymyxin A produced by *P. polymyxa* E681 contains Leu-Thr in the sixth and seventh positions, while commercialized polymyxins B and E have Phe-Leu and Leu-Leu, respectively, in these positions. The authors replaced the seventh L-threonine-specific A-domain (A_7-L-thr_-domain) region in polymyxin A synthetase genes with the A_7-L-leu_-domain region to construct polymyxin E synthetase genes. Additionally, the A_6-D-Leu_-domain region was substituted with the A_6-D-Phe_-domain region to generate polymyxin B synthetase genes. Production of the engineered polymyxins in surrogate hosts was confirmed by LC-MS analysis. Thus, heterologous expression of NRPS genes in easy-to-handle hosts, and domain swapping using marker-free genome editing technologies (Jeong et al., 2015; So et al., 2017), are promising routes for the development of new antibiotic derivatives.

### Fusaricidins

Fusaricidins produced by *P. polymyxa* strains are antibiotics in which six amino acids form a cyclic structure, and these compounds are highly active against Gram-positive bacteria and plant pathogenic fungi and oomycetes. Unlike other antibiotics made by *P. polymyxa*, fusaricidin activity was largely detected in the cell pellet extract, indicating association with the cell wall (Choi et al., 2008). The first fusaricidin biosynthetic gene was identified in *P. polymyxa* E681 as a single ORF encoding a NRPS with six modules (Choi et al., 2008). However, LC-MS analysis showed that strain E681 produced various types of fusaricidins, suggesting the NRPS could produce more than one type of fusaricidin, implying that the substrate-binding pockets of A-domains may be flexible and able to bind several types of amino acids. The second, fourth, and fifth modules of the NRPS have an E-domain that generates D-amino acids, but the sixth module has no E-domain, even though the sixth amino acid of all reported fusaricidins is D-alanine. It has since been demonstrated that the A-domain of the sixth module of fusaricidin NRPS directly activates the D-form of the amino acid (Li and Jensen, 2008).

Fusaricidin production has been successfully enhanced using traditional chemical mutagenesis, but regulation of fusaricidin production has been rarely reported (Kim et al., 2014). The two mutants, N1U7 and N17U7 (Figure 4) were generated and increased fusaricidin production 6.2- and 7.9-fold, respectively, than wild type strain. Whole genome sequencing of the mutants revealed a common mutation in the *pgm* gene that encodes an α-phosphoglucomutase. The *pgm* mutant strains exhibited reduced EPS production and increased cell growth and viability during stationary phase, which may be due to the increase in fusaricidin production in this mutant. In addition, RT-PCR analysis showed that the *pgm* mutation increased fusaricidin gene transcription fourfold. Further studies are needed to elucidate the mechanism of this transcriptional activation.

### Tridecaptins

Tridecaptins produced by *Paenibacillus* species are linear cationic lipopeptide antibiotics, including N-terminally acylated tridecapeptides. A gene cluster *trbABCDE* encoding tridecaptin synthetases was first identified in *P. polymyxa* E681 (Park et al., 2012a) and later in other *P. polymyxa* strains (Cochrane et al., 2015a). The *trbA* encodes a putative thioesterase, and *trbB* and *trbC* encode ABC transporters. TrbD and TrdE are NRPSs with 10 and 3 modules, respectively. Tridecaptins display strong antimicrobial activity against Gram-negative bacteria, including MRD strains of *K. pneumoniae* and *Acinetobacter baumannii*, by binding to the bacterial cell wall precursor lipid II on the inner membrane to disrupt the proton motive force (Cochrane et al., 2016). Due to low cytotoxicity and hemolytic activity, high stability in human plasma, and low levels of resistance development, tridecaptins are being studied as candidates for the treatment of MDR Gram-negative bacteria (Cochrane et al., 2014; Cochrane and Vederas, 2014). Since tridecaptins target the cell membrane, they can help other antibiotics to cross the membrane of Gram-negative bacteria in combination therapies. For example, tridecaptin has been covalently linked to other known antibiotics to enhance antimicrobial activity against Gram-negative bacteria, demonstrating great potential for effective treatment of Gram-negative pathogen infections (Cochrane et al., 2015b).

### Paenilipoheptin

Paenilipoheptin was recently identified by genome mining of *P. polymyxa* E681. Genome analysis of E681 revealed that the paenilipoheptin BGC is composed of five ORFs encoding the hybrid NRPS-*Trans*-AT-PKS (Vater et al., 2018). The first two ORFs (*phnAB*) encode *Trans*-AT-PKS, and the rest (*phnCDE*) encode NRPSs. PhnC, PhnD, and PhnE consist of one, one, and five modules, respectively. MALDI-TOF MS analysis revealed that a C12- or C13-β-amino fatty acid is linked via its amino group to the COOH group of the C-terminal L-Glu of paenilipoheptin. The antimicrobial activity spectrum remains to be elucidated.

### Paenilan

Paenilan, a ribosomally synthesized lantibiotic, was recently found in *P. polymyxa* E681 (Park et al., 2017). Analysis of the purified paenilan by nanoelectrospray ionization MS and tandem MS (MS/MS) revealed that it is a novel class I lantibiotic. The paenilan BGC spanning 13.2 kb comprises 11 ORFs. The prepeptide PnlA is predicted to consist of a 24 amino acid leader peptide and a 26 amino acid core peptide. A *P. polymyxa* E681 mutant (EPT14) lacking five BGCs for fusaricidin, polymyxin, tridecaptin, paenilipoheptin and uncharacterized polyketide (Figure 4) showed antimicrobial activity against Gram-positive bacteria such as *B. cereus*, *Micrococcus luteus*, and *P. durus*. The additional mutation in *pnl* BGC of EPT14 did not show antimicrobial activity against Gram-positive bacteria, indicating that the antimicrobial activity of EPT14 was derived from the paenilan.

### Other Antimicrobial Compounds

Genome analysis of *P. polymyxa* E681 using antiSMASH 4.0 revealed the presence of additional, yet uncharacterized, antibiotic BGCs, including *Trans*-AT-PKS-OtherKS-NRPS and lasso peptide gene clusters. The *Trans*-AT-PKS-OtherKS-NRPS genes (PPE_03009–PPE_03012) share 78% similarity with those of the bacillaene BGC of *B. amyloliquefaciens* FZB42 (Dunlap et al., 2016). The lasso peptide genes (PPE_05420) encode a putative class II bacteriocin, and 40% of genes in the cluster share similarity with those of the paeninodin BGC of *P. dendritiformis* C454.

## Industrial Implications

### Regulation of Sporulation

An in-depth understanding on the regulation of *P. polymyxa* sporulation is critical for developing industrial applications based on this strain, especially as continuous cultivation prohibits sporulation during the fermentation process. Sporulation initiation in *Bacillus* and related organisms including *Paenibacillus* spp. is dependent on a well-conserved multicomponent phosphorelay system. Multiple histidine kinases sense environmental sporulation signals and autophosphorylate themselves. The phosphate group of the histidine kinases is transferred to Spo0F, then to Spo0B, and finally to Spo0A. Phosphorylated Spo0A regulates the expression of genes related to sporulation. In general, Spo0F and Spo0A are highly conserved in *Bacillus* species, while Spo0B and the histidine kinases are less and poorly conserved, respectively. Spo0F and Spo0A of *P. polymyxa* E681 share 73 and 68% amino acid sequence identity, respectively, with those of *B. subtilis* 168, while Spo0B of E681 shares only 14% identity with *B. subtilis* Spo0B. The *P. polymyxa* E681 genome does not contain sporulation histidine kinase homologs to those in *B. subtilis*, although putative sporulation histidine kinase genes have been identified (Park et al., 2012e). Specifically, a histidine kinase (Kin1377) from *P. polymyxa* successfully restored sporulation in a sporulation-deficient *B. subtilis* mutant in which two major histidine kinase genes, *kinA* and *kinB*, were knocked out. The result suggested that the Kin1377 may be a sporulation histidine kinase in *P. polymyxa*. Another interesting sporulation histidine kinase (Kin1038) was also identified that partially restored sporulation in a *kinA* and *kinB* double mutant of *B. subtilis*. However, the additional introduction of the *P. polymyxa spo0A* gene into the mutant strain harboring Kin1038 induced significantly more spore formation. These results suggest that Kin1038 can bypass Spo0F and Spo0B, and transfer the phosphate group directly to Spo0A. A similar direct transfer of phosphate from histidine kinases to Spo0A was found in *Clostridium* species lacking *spo0F* or *spo0B* homologs in the genome (Worner et al., 2006).

### Active Inclusion Body Formation

Overexpression of recombinant proteins in *E. coli* often results in inclusion bodies in which misfolded proteins are aggregated and biologically inactive. This is commercially disadvantageous because solubilization of the inclusion bodies and refolding of the proteins are costly. Therefore, the formation of biologically active inclusion bodies in *E. coli* is attractive from a commercial perspective. *P. polymyxa* pyruvate oxidase (PoxB) forms large inclusion bodies when overexpressed in *E. coli* (Park et al., 2012d), which generates inclusion bodies with pyruvate oxidase activity. Fusion of GFP and *B. subtilis* AmyE to the PoxB also induced the formation of biologically active aggregates. The results indicate that PoxB can be used as a fusion partner for the formation of active inclusion bodies in *E. coli*.

### Boosting the Strength of Cement Paste

One interesting study demonstrated the industrial applicability of strain E681 (Park et al., 2014). Cement paste containing strain E681 displayed strong antifungal activity against *Aspergillus niger*, a deleterious fungus commonly found in cement buildings and structures. Strain E681 was also shown to help repair cracks by precipitation of calcium carbonate crystals in cement paste. These results suggest that strain E681 could be used in the development of multifunctional cement mortar.

## Conclusion

Herein, we summarized literature demonstrating that information derived from previous studies on *P. polymyxa* E681 provide an important gateway for promoting plant health and human healthcare. Strain E681 was initially studied as an outstanding PGPR based on its potent plant growth-promoting and plant-protecting activities, and bioinformatic and functional analyses of its genome subsequently illuminated various characteristics that benefit plants. More recently, strain E681 was found to be adept at producing various antibiotics, most notably polymyxins, recently re-instated as a therapy of last resort for treating Gram-negative infections. Other antibiotics produced by E681 include tridecaptin, paenilan, paenilipoheptin, and a putative polyketide, all of which are worthy of further study for the development of novel antibiotics against MDR human pathogenic bacteria. Fusaricidins elicit strong ISR effects, making them attractive as plant protectant agents in agriculture. A recombinant E681 strain that produces only fusaricidin at a high level by knocking out BGCs for other antimicrobial compounds has been reported.

We propose the need to reclassify sequenced strains in publicly available databases under the original designation of *P. polymyxa* species using genomic analysis of strain E681 and its close relatives. This could widen our genomic perspective on *P. polymyxa* and related species that have evolved under specific environmental niches, especially regarding their interactions with other organisms such as plants. In conclusion, bringing together up-to-date knowledge on the versatile *P. polymyxa* E681 strain could assist the development of highly functional microbial fertilizers and biocontrol agents, as well as industrial strains for the production of antibiotics and other valuable metabolites.

## Author Contributions

HJ conceived the project. HJ and S-HP sequenced the genomes and conducted comparative analysis. All authors wrote the manuscript and reviewed the final version of the manuscript prior to submission for publication.

## Conflict of Interest Statement

The authors declare that the research was conducted in the absence of any commercial or financial relationships that could be construed as a potential conflict of interest.

## Footnotes

1 http://www.bacterio.net/index.html, last update on 23 May 2018

2 www.kribb.re.kr

3 https://www.ncbi.nlm.nih.gov/genome/genomes/1386, as of 17 October, 2018
